# The effect of adding tobramycin to Simplex P cement on femoral stem micromotion as measured by radiostereometric analysis: A 2-year randomized controlled trial

**DOI:** 10.3109/17453674.2011.652885

**Published:** 2012-04-24

**Authors:** Eric Bohm, Martin Petrak, Trevor Gascoyne, Thomas Turgeon

**Affiliations:** ^1^Concordia Joint Replacement Group, Concordia Hip and Knee Institute; ^2^Division of Orthopaedic Surgery, University of Manitoba, Winnipeg, Manitoba, Canada

## Abstract

**Background:**

Previous in vitro research on addition of antibiotics to bone cement has found no statistically significant deterioration in mechanical properties. However, no clinical studies have compared the performance of tobramycin-laden bone cement with that of standard bone cement (Simplex P).

**Patients and Methods:**

23 patients (25 hips) were randomized to receive an Exeter (Stryker Orthopaedics) femoral stem cemented with either Simplex P (standard) or Simplex T (tobramycin-laden) cement. There were 2 years of follow-up, with scheduled radiostereometric (RSA) examinations.

**Results:**

All stems migrated distally and showed some degree of retroversion. No clinically significant differences in stem subsidence or retroversion were found between the Simplex T and Simplex P cement groups after 2 years. Overall subsidence was less than in previous studies, probably due to a postponed initial post-surgical examination. Rates of subsidence in both cement groups were consistent with those from previous studies of Exeter stems.

**Interpretation:**

Subsidence of the femoral stem after 2 years was similar in the Simplex T (tobramycin-laden) and Simplex P (standard) groups.

When antibiotic-laden bone cement (ABLC) was introduced in 1970, there were concerns that the addition of antibiotic powder to bone cement could compromise the mechanical properties of the cement, and therefore increase the risk of aseptic loosening of arthoplasty components (Murray 1984, Lundberg and Hedlund 2007). Since then, numerous in vitro studies have shown that the addition of less than 2 g of antibiotic to 40 g cement powder has a negligible effect on the mechanical strength and fixation properties of bone cements (Davies and Harris 1991, Klekamp et al. 1999, Bourne 2004). However, there is some debate about the appropriateness of laboratory testing of cement properties (Nottrott et al. 2008) since regulatory standards require that bone cement be tested after 24 h of ageing under dry conditions at 23°C (an environment very unlike that of the human body), and not over an extended time (Nottrott et al. 2008). Furthermore, success in the laboratory does not guarantee long-term clinical success, as seen with the disastrous outcomes with Boneloc cement (Gebuhr et al. 2000). This underscores the need for rigorous clinical testing and in vivo measurements of new products prior to adopting them for routine use (Thanner et al. 1995, Hallan et al. 2006).

Large cohort studies have shown that the prophylactic use of ABLC is associated with a lower risk of infection-based revision (Havelin et al. 1995, Engesæter et al. 2003). Unfortunately, such studies may have underestimated the true aseptic loosening-based failure rates, as some patients with loose implants may never undergo revision (Soderman et al. 2001). Radiostereometric analysis (RSA) has also been used to investigate the performance of new antibiotic-laden cements (Adalberth et al. 2002, Hallan et al. 2006). Hallan et al. (2006) compared the extent and patterns of migration between Charnley total hips randomly cemented with either Refobacin-loaded Palamed G or gentamicin-loaded Palacos R, and found similar migration patterns. Such studies are useful for comparing different ABLCs, but studies that directly examine the effect of adding antibiotic to a specific cement on the risk aseptic loosening are needed. Thus, we determined whether the addition of tobramycin to Simplex P cement increases the risk of long-term aseptic loosening as predicted by implant micromotion detected by RSA. We selected tobramycin-laden Simplex cement (Simplex T), as it was a relatively new ABLC introduced into North America at the start of this study in 2003.

## Patients and methods

We conducted a triple-blind, randomized controlled trial to answer the study question. Inclusion criteria were patients over the age of 60 undergoing a primary total hip arthroplasty. Exclusion criteria included post-traumatic arthritis, rheumatoid arthritis, hip dysplasia, previous hip infection, or renal insufficiency defined as a creatinine level of > 130 µmol/L (due to the theoretical risk of nephrotixicity associated with tobramicin). Patients were recruited by a study coordinator from the elective practice of an academic arthroplasty group. Operations were performed at Concordia Hospital, Winnipeg, Canada, with all pre- and postoperative visits taking place in the surgeon’s clinic. Physical examinations of patients were performed by the surgeon, while the functional questionnaires were collected by the study coordinator. Approval of this study was granted by the University of Manitoba Research Ethics Board on July 15, 2003 (BREB# B2003:108) in compliance with the Helsinki Declaration (1975). Informed consent was obtained for each patient enrolled in the study.

### Surgical procedure

The 4 surgeons who performed the procedures were all fellowship-trained. Direct lateral or posterior approaches were used. Hip prostheses consisted of Exeter femoral stems coupled with Trident acetabular cups (Stryker Orthopaedics, Mahwah, NJ). The Exeter stem was used because it has excellent long-term survival, and it is available with tantalum markers on the shoulder and tip to facilitate marker-based radiostereometric analysis (Carrington et al. 2009).

Randomization to cement type occurred in the operating room using sequential envelope selection, ensuring the surgeon was blinded to the type of cement. The random allocation sequence was determined with permuted blocks of 4 using a table of randomized numbers. The surgeon, patient, research co-ordinator, and engineers performing the analysis were all blinded regarding the cement type. A third-generation cementing technique was used (vacuum mixing, brushing, lavaging, and plugging of the femoral canal, and retrograde insertion with pressurization) for standardized times as recommended by the manufacturer.

Tantalum beads 1 mm in diameter were injected into the greater trochanter, the lesser trochanter, and the femoral shaft distal to the tip of the stem during surgery. Patients received 1 preoperative dose of antibiotics and another 24 h postoperatively. Low-molecular-weight heparin was given for 28 days. Patients were weight bearing as tolerated immediately after surgery, and they followed a standardized physiotherapy protocol.

### Radiostereometric analysis

RSA examinations were planned at 6 weeks, 6 months, 1 year, and 2 years postoperatively. The 6-week examination served as baseline. RSA radiographs were obtained from patients lying in the supine position above a uniplanar calibration cage (Cage 43; RSA Biomedical, Umeå, Sweden). The 2 X-ray sources included a fixed ceiling-mounted tube (Philips Medio 50 CP) and a mobile tube (Shimadzu MobileArt Plus) positioned at 40˚ to each other, 140 cm above the films/cassettes. Initially, conventional radiographic films (35 × 43 cm) were developed using conventional radiography for eight examinations, but were eventually replaced by high-resolution CR cassettes (AGFA MD4.0, 35 × 43 cm) for the majority of the examinations. The films were scanned with a Umax PowerLook 2100XL flatbed scanner (UMAX Technologies, Dallas, TX) and the CR cassettes were digitized with an AGFA ID Tablet system.

Double examinations were performed in at least 1 follow-up for each patient, in order to obtain the precision (i.e. the detection limit) of the RSA system (Valstar et al. 2005, Derbyshire et al. 2009). Precision was calculated by multiplying the standard deviation of absolute migration differences by the corresponding Student’s t-value (Derbyshire et al. 2009).

Radiographic measurements and analyses were performed with the UmRSA software suite version 6.0 (RSA Biomedical, Umeå, Sweden). The tantalum markers were used together with femoral head edge-detection algorithms to determine the migration of the stem relative to the bone at each of the follow-up periods. The maximum allowable condition number for rigid body fitting of the bone and femoral stem was set at 90 with a mean error of 0.2 mm. Also, the maximum allowable distance between crossing lines was set at 0.3 mm (Valstar et al. 2005).

### Assessment

The primary outcome metric was stem subsidence as measured with RSA; secondary measures included stem rotation and functional scores. Functional assessment was performed using the Harris Hip Score and Western Ontario and McMaster Universities Arthritis Index score.

Analysis of motion was performed on the centroid of the implant for each individual axis of translation and rotation. The centroid was determined using 3 fixed points: a marker on the distal tip of the stem, a marker at the shoulder, and the femoral head center as determined by edge-detection algorithms of the UmRSA program. Analysis of the direction of total implant movement was performed in the manner recommended by Derbyshire et al. (2009). Calculation of the rate of subsidence was done by averaging the slopes of the linear regression lines for each patient in each cement group (McCalden et al. 2009). Migration rates were used to compare our findings with those of other studies, since rates are not dependent on the timing of the initial reference examination (6 weeks vs. immediately postoperatively).

### Statistics

The study was powered to detect whether or not a clinically significant difference in stem subsidence existed between the Simplex T and Simplex P groups. Based on the work of Kobayashi et al. (1997) and Alfaro-Adrian et al. (1999), we set the minimum clinically significant difference in subsidence rates at 2 years that would predict a higher failure rate in Simplex T cement at 0.4 mm. We chose a standard deviation of 0.35 mm, which is consistent with the standard deviation of the 2-year measurements in this study as well as that used by others (Glyn-Jones et al. 2006). This provided a sample size of 13 hips per group at a significance level of 5% and a power of 80%. To allow for dropout or loss to follow-up, the recruitment size was increased to include a minimum of 33 subjects.

Means, standard deviations, and 95% confidence intervals were calculated for the migration data. A Kolmogorov-Smirnov goodness-of-fit test for normal distribution was performed with SPSS software version 17.0. Statistical comparison of the RSA and clinical results was performed using an unpaired Student’s t-test. Significance was set at p < 0.05.

## Results

### Participants

**Figure 1.**
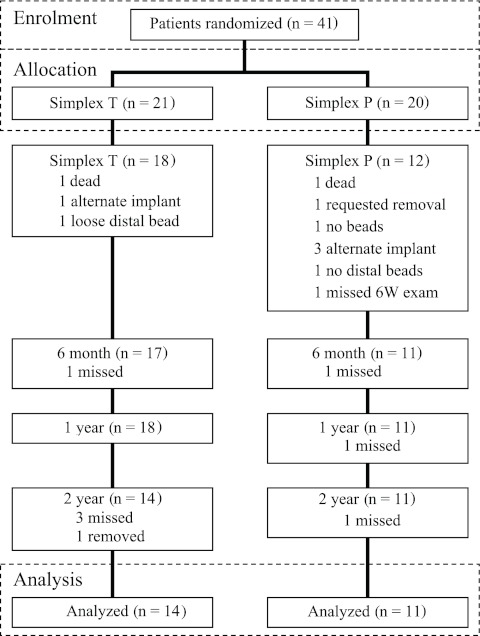
Participant flow.

38 patients (41 hips) were initially recruited for this study between March 2004 and June 2007; however, only 28 patients (30 hips) were included in the follow-up due to initial patient dropout (Figure 1). These 28 patients consisted of 18 females (2 bilateral) and 10 males, with an average age at time of surgery of 73 (range: 63–85) years. The Simplex T group consisted of 16 patients (18 hips) with a mean age of 73, and the Simplex P group consisted of the remaining 12 patients (12 hips) with a mean age of 72. Due to missed examinations and further patient dropout, 23 patients (25 hips) were used for the 2-year analysis.

Implant position relative to the femur was measured at each follow-up period, but actual follow-up times varied slightly. The mean follow-up times were 7.0 (1.3–15.3) weeks for the 6-week follow-up, 6.2 (5.1–7.5) months for the 6 month follow-up, 12 (11–14) months for the 1-year follow-up, and 24 (23–27) months for the 2-year follow-up.

### Clinical assessment

No statistically significant differences in clinical scores were found between the Simplex-T cement group and the Simplex-P group, either preoperatively or during any of the follow-up visits (Table 1, see supplementary data). No revisions or dislocations occurred during the 2-year period.

### Radiostereometric analysis

The mean UmRSA condition number was 45 (21–84) with a mean error of 0.120 (0.027–0.203) mm for the femur, and 39 (33–48) with 0.072 (0.019–0.189) mm mean error for the implant centroid. The precision of the RSA system in the measurement of implant migration is summarized in Table 2. Translational error was greatest along the anterior (z-) axis, which is consistent with findings in previous studies (Alfaro-Adrian et al. 1999).

**Table 2.** Precision of the RSA system in measurement of migration of the femoral stem centroid, based on 49 double examinations. The x-, y-, and z-axes correspond to the medial, proximal, and anterior axes, respectively

	Translation	Rotation
Axis	Mean absolute error	Mean absolute error
	(95% CI), mm	(95% CI), degrees
x	0.030 (+/- 0.092)	0.079 (+/- 0.200)
y	0.035 (+/- 0.087)	0.163 (+/- 0.501)
z	0.061 (+/- 0.157)	0.027 (+/- 0.079)

**Figure 2.**
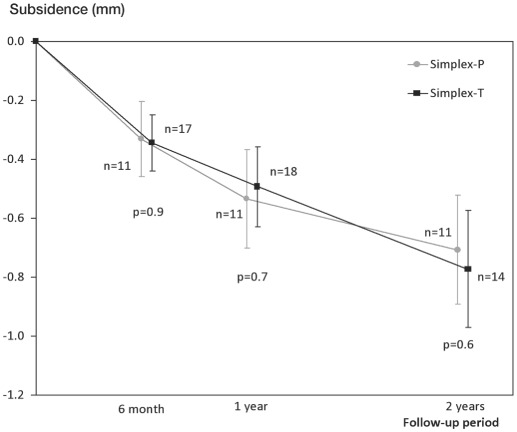
Mean subsidence of the stem centroid. Error bars represent the 95% confidence interval. Sample size (n) and p-values are shown for each group at each follow-up period.

After 2 years, mean subsidence of the femoral stem was 0.71 mm (SD 0.30) in the Simplex P group and 0.77 mm (SD 0.36) in Simplex T group (Figure 2). The difference in stem subsidence between the 2 groups was 0.06 mm, with the 95% confidence interval of this difference ranging from –0.21 to 0.34 mm (Table 3).

**Table 3.** Two-year femoral component micromotion and differences for each cement group

	Simplex P		Simplex T		Difference	95% CI of the difference	p-value
Axis	Mean	SD	Mean	SD	(SimP–SimT)		
Translation (mm)							
x_T_: medial+ / lateral–	–0.01	0.09	0.01	0.13	–0.02	–0.11 to 0.08	0.8
y_T_: proximal+ / distal–	–0.71	0.30	–0.77	0.36	0.06	–0.21 to 0.34	0.6
z_T_: anterior+ / posterior–	–0.09	0.21	–0.17	0.23	0.08	–0.10 to 0.26	0.4
Rotation (degree)							
x_R_: anterior+ / posterior– tilt	0.01	0.20	–0.11	0.17	0.12	–0.03 to 0.28	0.1
y_R_: retroversion+ / anteversion–	0.60	0.73	0.84	0.93	–0.24	–0.94 to 0.47	0.5
z_R_: valgus+ / varus– tilt	0.00	0.11	–0.04	0.12	0.04	–0.06 to 0.13	0.4

**Figure 3.**
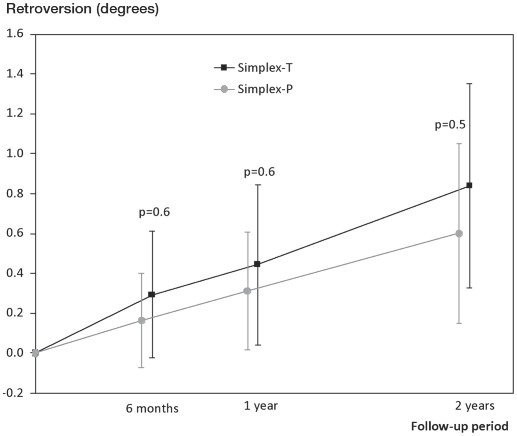
Progression of mean retroversion of the stem centroid with each follow-up period. Error bars represent the 95% confidence interval. P-values are shown at each follow-up period

Stem retroversion was the main direction of rotation for both cement groups. After 2 years, the mean retroversion was 0.60˚ (SD 0.72) and 0.84˚ (SD 0.93) for the Simplex P and Simplex T groups, respectively (Figure 3).

Analysis of stem subsidence between the first- and second-year follow-up periods revealed a small but statistically insignificant difference in subsidence rates between the cement groups: 0.16 mm/year (SD 0.10) for Simplex-P and 0.24 mm/year (SD 0.13) for Simplex T (p = 0.08). Linear regression was used to represent the rate of stem subsidence, since it is expected to be relatively constant after an initial bedding-in period, which was selected to be 1 year (Waanders et al. 2009).

As recommended by Derbyshire et al. (2009), the cumulative second-year migrations of the stems were plotted in the coronal and sagittal planes. Two distinct trends were visible: substantial distal migration of the stem and moderate posterior migration, which were similar in both groups (Figure 4, see supplementary data).

One bilateral patient in the Simplex T group had subsidence of more than 2 SD greater than the mean: 1.44 mm (left hip) and 1.43 mm (right hip), compared to the Simplex T group mean of 0.77 mm. The data were re-examined with this patient removed. This resulted in the mean subsidence of the Simplex T group decreasing to 0.66 (SD 0.24) mm from 0.77 (SD 0.36) mm. The mean retroversion for the Simplex T group diminished from 0.84° (SD 0.93) to 0.70° (SD 0.76) after removal of this patient. Furthermore, the mean rate of subsidence between the first and second follow-up was found to be 0.20 mm/year, which was closer to the Simplex P rate of 0.16 mm/year (p = 0.2). This patient will be watched closely in the future for evidence of early loosening.

## Discussion

The main study question was whether or not the addition of tobramycin to Simplex P bone cement increases the risk of long-term aseptic loosening as predicted by stem subsidence measured by RSA. We found that the difference in stem subsidence between the cement groups at 2 years was 0.06 mm (95% CI: –0.21 to 0.34). This difference was smaller than the clinically important difference of 0.4 mm, and therefore confirms that there was equivalence between the 2 cement types.

We found a difference of 0.24° (95% CI: –0.94 to 0.47) in stem rotation between the 2 cement types. This difference was not clinically or statistically significant, as the standard deviations were larger than the means for both cement groups.

This study was powered to detect differences in stem subsidence, and as expected, we were unable to detect any differences in clinical outcome scores between the groups.

The subsidence measured for the Exeter stems was less than in most other studies with these stems. For instance, previous studies have reported 2-year subsidence of 0.92 mm (Glyn-Jones et al. 2006), 1.07 mm (Glyn-Jones et al. 2003), 1.20 mm (Alfaro-Adrian et al. 1999), 1.34 mm (Stefansdottir et al. 2004), and 1.35 mm (McCalden et al. 2010)—all of which are substantially higher than the 2-year subsidence figures of 0.71 mm for Simplex P and 0.77 mm for Simplex T in the present study. This difference is probably due to the variation in timing of the reference examination; we chose to perform ours at 6 weeks postoperatively, whereas most others chose to perform theirs within 1 week of surgery (Ornstein et al. 2000). Accordingly, it is more appropriate to compare our findings to published migration and rotation rates between the first- and second-year follow-up periods, since these values do not depend on the timing of the initial reference examination (Derbyshire et al. 2009).

3 previous studies have determined mean subsidence rates for the Exeter stem between the first-year and second-year follow-up examinations: ∼0.11 mm/year (Stefansdottir et al. 2004), 0.14 mm/year (Alfaro-Adrian et al. 1999), and 0.2 mm/year (Alfaro-Adrian et al. 2001). These rates are fairly consistent with our mean subsidence rates for the Simplex P and Simplex T cement groups of 0.16 mm/year and 0.24 mm/year.

Retroversion of the Exeter stems in our study was 0.60° and 0.84° after 2 years in the Simplex P and Simplex T groups, respectively. These findings are somewhat lower than in 2 previous studies, with a mean of 1.1° at 2 years (Alfaro-Adrian et al. 2001) and a median of 1.2° at 2 years (Stefansdottir et al. 2004). However, as mentioned above, this variation can be attributed to differences in timing of the reference RSA examination.

### Limitations

The Exeter femoral stems used in the present study were implanted with both the lateral and posterior surgical approaches. Glyn-Jones et al. (2006) compared the effect of surgical approach on migration of Exeter stems, and found that stems inserted with the posterior approach showed statistically significantly greater amounts of retroversion: 1.16° (lateral technique) and 1.94° (posterior technique). In our study, the posterior approach was used in only 3 hips in the Simplex-P group and 2 hips in the Simplex-T group. Thus, this would not be expected to have confounded the results. Even so, there should be consistency in the surgical approach in future studies.

Our experimental precision values of 0.087–0.157 mm (translation) and 0.079–0.501° (rotation) are comparable to or better than those in previous RSA studies (Alfaro-Adrian et al. 1999, Ornstein et al. 2000, Glyn-Jones et al. 2003, 2006). 3 patients were lost from the Simplex P group due to the use of an alternative implant. This could have introduced selection bias into our results.

All hips examined were treated as independent samples; however, 2 bilateral patients were included in the final analysis of stem subsidence and stem rotation, which violates the statistical independence of our samples (Bryant et al. 2006, Ranstam 2009). However, this bias would not be expected to be significant, as RSA is a precise and objective measurement technique and is therefore unlikely to be affected by duplicate patients. Also, we analyzed the data after removing both of these bilateral patients and it made no difference to our findings.

To our knowledge, this is the only study to have provided a direct measure of the in vivo effect of adding tobramycin to bone cement on micromotion of femoral hip prostheses. Our findings are consistent with the findings of other studies on migration of the Exeter stem, concerning both distal migration and retroversion. This study also confirms the findings of previous clinical and experimental studies that could only indirectly and partially assess the effect of antibiotic addition on the risk of aseptic loosening: addition of tobramycin antibiotic to Simplex P cement in small amounts does not appear to increase the risk of aseptic loosening, as predicted by in vivo subsidence. Further follow-up (of more than 5 years) will be done to assess the long-term results.

## Supplementary data

Table 1 and Figure 4 are available at the website (www.actaorthop.org), identification number 4640.
