# Supplementary material for: The effect of adding tobramycin to Simplex P cement on femoral stem micromotion as measured by radiostereometric analysis: A 2-year randomized controlled trial
Source: Acta Orthop. 2012 Apr 24;83(2):115–20. doi: 10.3109/17453674.2011.652885 (PMC3339523; doi:10.3109/17453674.2011.652885)
Supplement: Supplementary file 1 [file ORT-1745-3674-83-115-s4640.pdf]

Supplementary article data

The effect of adding tobramycin to Simplex P cement on femoral stem micromotion as measured by radiostereometric analysis

A 2-year randomized controlled trial

Eric Bohm[1,2], Martin Petrak[1], Trevor Gascoyne[1], and Thomas Turgeon[1,2]

[1]Concordia Joint Replacement Group, Concordia Hip and Knee Institute; [2]Division of Orthopaedic Surgery, University of Manitoba, Winnipeg, Manitoba, Canada
Correspondence: ebohm@cjrg.ca
Submitted 11-01-25. Accepted 11-10-09

Table 1. Outcome measures for the two cement groups. WOMAC and Harris hip scores for pre- and post-operative follow-ups. Values are mean (range)

	Harris hip score			*WOMAC score*		
	Simplex-T	*Simplex-P*	*p-value*	*Simplex-T*	*Simplex-P*	*p-value*
Preoperatively	*47 (30–80)*	*50 (22–70)*	*0.7*	*43 (7–94)*	*51 (24–76)*	*0.2*
6 months	*77 (26–100)*	*82 (50–97)*	*0.5*	*85 (57–100)*	*85 (53–100)*	*0.9*
1 year	*83 (49–96)*	*80 (31–100)*	*0.7*	*87 (58–100)*	*83 (26–100)*	*0.6*
2 years	*86 (58–100)*	*88 (67–100)*	*0.8*	*88 (66–100)*	*94 (81–100)*	*0.2*

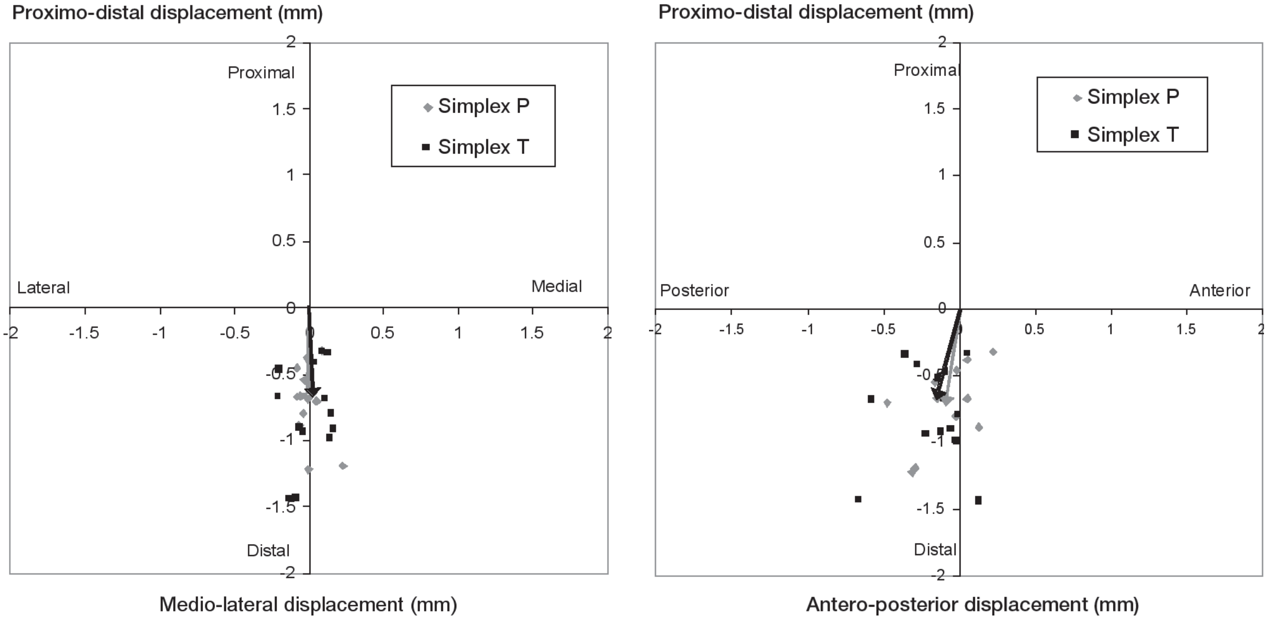

Figure 4. Scatter-plots of 2 year migrations of individual stems in the coronal plane (left panel) and sagittal plane (right panel) for both cement groups. Arrows indicate the mean direction and amplitude of migration.

Open Access - This article is distributed under the terms of the Creative Commons Attribution Noncommercial License which permits any noncommercial use, distribution, and reproduction in any medium, provided the source is credited.
DOI 10.3109/17453674.2011.652885
